# Phosphorus Use Efficiency of Leafy *Brassica* sp. Grown in Three Contrasting Soils: Growth, Enzyme Activity and Phosphorus Fractionation

**DOI:** 10.3390/plants12061295

**Published:** 2023-03-13

**Authors:** Branimir Urlić, Gvozden Dumičić, Tomislav Radić, Smiljana Goreta Ban, Marija Romić

**Affiliations:** 1 Department of Applied Science, Institute for Adriatic Crops and Karst Reclamation, Put Duilova 11, HR-21000 Split, Croatia; 2 Institute of Agriculture and Tourism, K. Huguesa 8, HR-52440 Poreč, Croatia; 3 Centre of Excellence for Biodiversity and Molecular Plant Breeding, HR-10000 Zagreb, Croatia; 4 Faculty of Agriculture, University of Zagreb, Svetošimunska 25, HR-10000 Zagreb, Croatia

**Keywords:** kale, soil P fractions, phosphatase, P uptake and utilization efficiency

## Abstract

Plant adaptations to low soil phosphorus (P) availability have been intensively studied in *Brassica* sp. in an attempt to identify the mechanisms involved in P uptake and utilization. The present pot experiment was conducted to evaluate the relationships between plant shoot and root growth, P uptake and use efficiency parameters, and P fractions and enzyme activity, in two species grown in three soil types. The aim of this study was to determine whether adaptation mechanisms are soil-dependent. Two kale species were grown in soils typical for coastal Croatia (terra rossa, rendzina, and fluvisol) with low P availability. Plants grown in fluvisol had the highest shoot biomass and accumulated most P, whereas plants developed the longest roots in terra rossa. Phosphatase activity differed among soils. P use efficiency differed among soils and species. Genotype IJK 17 showed better adaptation to low P availability, which was related to better uptake efficiency. In general, soils differed in inorganic and organic P fractions in rhizosphere soil, but no difference between genotypes was found. The activities of alkaline phosphatase and phosphodiesterase were negatively correlated with most organic P fractions, suggesting their function in the mineralization of soil organic P. Kale species activate different mechanisms of P uptake and utilization when grown in contrasting soil types, suggesting that specific responses to the soil type were more important than the genotypic difference.

## 1. Introduction

Phosphorus (P) is one of the essential elements for plant growth, but in both natural and agricultural systems its limited availability often hampers productivity. The problem of P deficiency on a global scale becomes particularly important due to problems with exploitation of phosphate reserves and an insufficient use of expensive and unaffordable P fertilizers [1]. Although many soils have high total P concentrations, the concentration of P in soil solution is very low, ranging between 0.1 and 10 μM [2], and is suboptimal for plant requirements.

Phosphorus has low mobility and high fixation to soil constituents, with the formation of poorly soluble Ca phosphates in neutral/calcareous soils or adsorption by Al/Fe oxide and hydroxides in acidic soils. Moreover, organic P constitutes between 20% and 80% of the total P in soils as a part of organic complexes [3]. Plants release phosphatases from roots under low P, allowing the mobilization of organic P in soil [4]. P fractionation is an effective tool to investigate soil P availability and possible conversion among soil inorganic and organic fractions. In sequential P fractionation, the strength of the extracting solution increases stepwise, in order to separate more strongly bound P forms [5,6]. Redistribution among various fractions is important for P mobility, as more P in labile pools indicates greater potential availability to plants [7].

Plant species and genotypes differ in their capacity to tolerate low P availability. Through evolution, plants evolved adaptive morphological, physiological, and biochemical mechanisms to access P under limiting conditions. These include the modification of root architecture by increasing the exploited soil volume, increased organic acid anion excretion and rhizosphere acidification, enhanced phosphatase and phytase production, and the promoted expression of P transporter genes [8,9].

Phosphorus use efficiency (PUE) can be defined as the amount of plant biomass or yield produced per unit of P taken up. There are numerous definitions and calculation methods used for the interpretation of nutrient use efficiency, making the comparison of different results and experiments difficult. PUE has two components: P uptake/acquisition efficiency (PUpE) and P utilization efficiency (PUtE). Acquisition efficiency represents a plant’s capacity to take up P from growing media, whereas utilization efficiency is defined as a plant’s capacity to produce biomass or economic yield with an amount of P present in biomass [10]. Higher PUE can be achieved by improving one of these components or both.

Kales (un-headed *Brassica* species) are the oldest cultivated species from the *Brassicaceae* family with a long history of use that has resulted in a large number of kale genotypes showing huge genetic variability. *Brassica* growth and development are considered to be more effective than other plant families at low P availability due to the solubilization of Ca-P, the exudation of organic acid anions, or modification in root architecture, and they can effectively use P from organic and inorganic sources [11,12]. Kales are widely grown in Croatia, mostly on soils with low levels of plant-available P. The screening for PUE parameters of Croatian kale genotypes was conducted in a soilless system and showed high PUE variability [13].

Some of the widespread P-deficient soils in Mediterranean/coastal Croatia include rendzina, terra rossa, and fluvisols. Rendzina (RZ) is developed on limestone, marls, or other carbonate rich parent materials. It is characterized by the occurrence of limestone rock fragments, optimal drainage with a basic reaction, and a high content of CaCO_3_, and is formed by the weathering of carbonate rocks. Rendzina’s soil cover is erratic and disrupted by bare limestones and karst forms. Terra rossa (TR) is a non-calcareous soil with a slightly acid or neutral reaction and a near-saturated adsorbing complex. It is a reddish clay to silty–clay soil, and is widespread in the Mediterranean, where it covers limestone and dolomite with different thicknesses [14]. Fluvisols (FL) are young soils characterized by favorable physicochemical properties that originate from alluvial deposits [15,16].

The aim of this study was to compare the growth, P uptake, and use efficiency of two *Brassica* genotypes in three contrasting soils with low P availability. Additionally, for the first time, on the same soils complete P fractionation was performed and the fractions content was compared with plant growth, P uptake and utilization traits. This study should provide a better understanding of whether adaptation mechanisms to soil P deficiency change with cultivation in different soils, as well as identify the reaction of leafy *Brassica* genotypes.

## 2. Results

### 2.1. Plant Growth and P Accumulation

The shoot DW, leaf area, and number of leaves were significantly (*p* < 0.001) higher in plants grown on FL soil compared to plants grown on TR and RZ soils (Table 1). No significant interactions between soils and genotypes were found. However, the opposite trend was found for the root:shoot ratio, where a lower ratio was determined in plants grown on FL (*p* < 0.001), and genotype IJK17 had a lower ratio than RR (*p* < 0.01). Shoots and roots had significantly higher P concentrations (*p* < 0.001) on FL soil, and this was related to the biomass accumulated; plants on this soil had the highest P content in shoots (*p* < 0.001) and roots (*p* < 0.01).

Plants grown on TR soil had significantly longer roots (*p* < 0.05) than plants grown on other soils (Table 2); the same was found for root surface area and volume (data not shown). Moreover, TR soil plants had a larger root diameter than RZ soil plants (*p* < 0.01). Genotypes did not show differences regarding root traits or P accumulation parameters.

Specific root length (SRL) was highest in TR and significantly differed only from FL soil (*p* < 0.001). Regarding SPU, which shows plant P uptake per unit of root length, the highest values were found in FL (*p* < 0.001), and were four–fold higher than in TR and RZ soils.

No differences were found between soils for acid phosphomonoesterase (AcPe) activity, although the highest activity was noted for TR (Table 2). RZ and FL soils had significantly higher alkaline phosphomonoesterase (AlPe) activity (*p* < 0.001) compared to TR. Phosphodiesterase (DPe) activity in all soils differed from each other (*p* < 0.001) with the highest activity in RZ > FL > TR.

### 2.2. P use Efficiency Parameters

The highest PUpE values were found in plants grown in RZ soil and were significantly higher (3–4 fold) than in TR and FL (Table 3). RZ soil also had the highest PutE. This was not statistically different from TR, but both differed from FL. PUE was obtained as a multiplicative value from PUpE and PUtE, and differed between all soils. It was highest in RZ and lowest in FL soil. Genotype IJK 17 was significantly more efficient in terms of PUE than RR, while the soil×genotype interaction was not significant, so it was not shown in tables.(

The relative contribution of PUpE and PUtE to variations in PUE is shown in Table 4. This method showed great variability between soils regarding the contribution of PUpE and PUtE to biomass production (as shoot DW). In TR soil, PUE variability could be highly explained (96%) by PUpE variation, while in RZ, PUpE was also important, but to a lesser extent (65%). The higher contribution of PUtE to PUE variability (60%) was noted for FL soil. Both genotypes had a greater PUpE influence on PUE compared to PUtE.

### 2.3. Soil P Fractions

**Figure 1:**
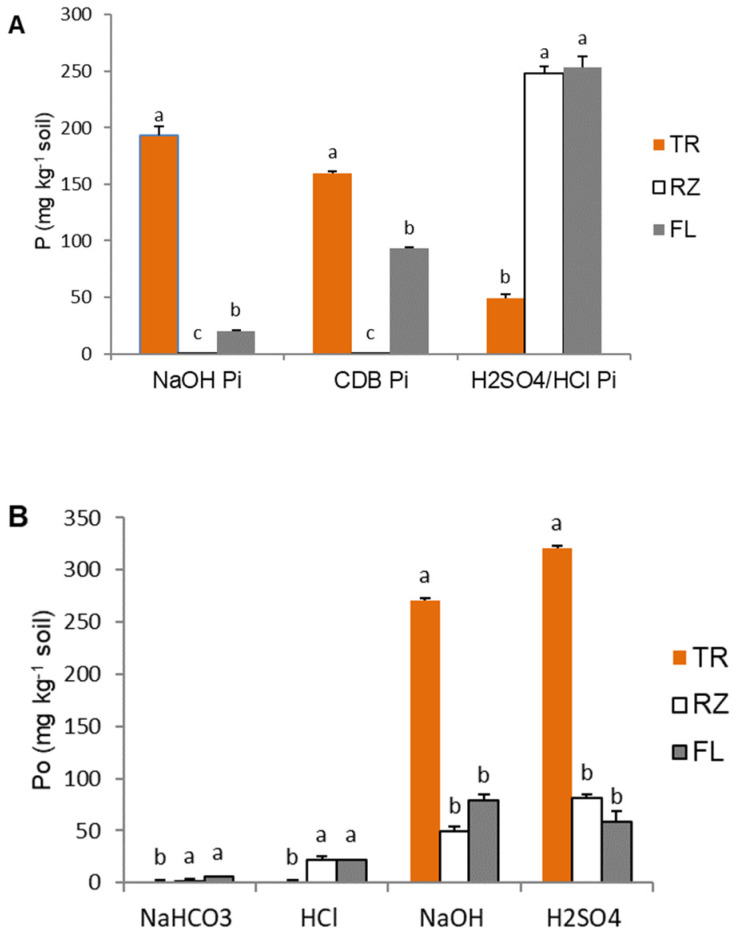
Inorganic-Pi (**A**) and organic-Po (**B**) fractions in the rhizosphere soils of two *Brassica* genotypes. TR, RZ, and FL represent soils Terra rossa, Rendzina and Fluvisol, respectively. Within a given P fraction, bars with the same letter are not significantly different according to Tukey HSD test. Error bars represent standard error (±1 SE).

Generally, soils significantly differed in total inorganic (Pi) and organic P (Po) from rhizosphere soil (Figure 1). The m concentration values decreased in the order TR > FL > RZ for both parameters. No difference between genotypes was found for any P fraction or total P amount. The soluble bound fractions extracted with NH_4_Cl and NH_4_F were not detected in TR soil, so these data are not shown in Figure 1A. The fractions NaOH Pi (Fe-P) and CBD Pi (reductant soluble Pi) were highest in TR, while the same fractions (labile and reductant) were not detected in RZ soil (Figure 1A). Soils FL and RZ had higher H_2_SO_4_ (HCl) Pi than in TR.

For organic fractions, easily available NaHCO_3_ Po content was the highest in FL, but was not significantly different to RZ (Figure 1B). The same was found for moderately available HCl Po. Both fractions were under the detection limit in TR soil. Humic NaOH Po was significantly higher in TR compared to FL andRZ soil. Non-labile (residual) H_2_SO_4_-P_o_ concentration in TR soil differed from the other two soils, but differences were not found between them.

Significant correlations between shoot P concentrations and each fraction were only found for non-labile Po (r = −0.43; *p* ≤ 0.05). For soils separately, significant correlations were only determined in RZ soil for labile (r = −0.73; *p* ≤ 0.05) and non-labile Po (r = −0.88; *p* ≤ 0.01). The correlations between the fractions in all soils together were not determined, and separately only in TR. Inorganic reductant soluble CBD Pi correlated with HCl Pi (r = 0.80; *p* ≤ 0.05), humic P_o_ (r = 0.85; *p* ≤ 0.01), and non-labile Po (r = 0.82; *p* ≤ 0.01) and a correlation was also found between humic Po and non-labile Po (r = 0.82; *p* ≤ 0.05).

Correlations between enzymatic activity and organic P fractions (Po) were calculated for all soils and genotypes together (Table 5). A positive significant correlation was noted between AlPe and Dpe (*r^2^* = 0.75). Both of these enzymes negatively correlated with humic (NaOH) Po, residual/nonlabile (H_2_SO_4_) Po, and total Po. Significant correlations were found between enzymes and moderately labile HCl Po: negative for AcPe and positive for AlPe and Dpe.

## 3. Discussion

The plants of both genotypes had best shoot growth with more leaves and higher leaf area in FL and the worst in RZ, which is likely related to concentrations of available soil P (Table 1). The available soil P, especially for TR and RZ, was much lower than 100 mg P_2_0_5_/kg, which is a critical value for the category of very low P availability, according to the Troug method (extraction with ammonium-sulphate) [17]. The shoot P concentrations in plants grown on all soils were lower than those noted by Hammond et al. [18] for *Brassica oleracea* genotypes grown in optimal P conditions. Taking into account the deficiency limit of 2 mg g^−1^ DW, our results showed P deficiency in TR and RZ, while plants from FL had marginal leaf P concentration.

The highest P accumulation in the roots and shoots of plants grown in our experiment was in FL. Unlike FL, which resulted in the best shoot biomass, TR and RZ soils with low concentrations of plant available P resulted in more intensive root development (Table 2). Thus, roots in these two P types of soil with low P availability accumulated 13–16% of total plant P, which was significantly higher compared to FL. This root adaptation to low P falls into a wide range of responses of root architecture development to low P availability [19,20]. The average root diameter was highest in TR, indicating preferred secondary/lateral root growth. Moreover, in the same soil (TR), roots were significantly longer than in the two other soils. This can be linked to root elongation (etiolation) mechanisms, as many *Brassica* species respond by thickening primary roots and investing biomass in both lateral roots and root hairs under low P availability. An increased distribution of *Brassica napus* coarse roots in surface soil improved P uptake [21]. It seems that both mechanisms (elongation and lateral growth) were involved. Lateral growth was mostly higher under P deficiency in a large screening study of *B. oleracea* accessions [18], as well as in white lupin under acidic and neutral conditions with most of the roots belonging to thin roots [22]. As was found in maize grown on neutral soil or the above mentioned white lupin, root diameter should decrease at low P availability compared to alkaline soil [22,23]; a possible explanation could be increased secondary growth, and at the same time etiolation as a process that requires a lower metabolic cost [24].

In this study, specific root length (SRL) was higher in soils with lower Pi concentrations, TR and RZ, and root: shoot ratio was higher than in FL, indicating plant reaction on a low P supply (Table 2). SRL is a relevant parameter, as it defines soil volume in contact with roots due to preferred allocation of photosynthates to roots, which refers to the lower cost of organic C under low soil P [8]. Similarly, SRL was increased in *B. napus,* maize or white lupin when grown in acidic soil with low P, as in TR in our study [22,23,25]. Specific P uptake (SPU) showed that plants from FL had greater capacity for P uptake regarding root length, which assumes better P availability in this soil [10].

The shoot P content in our study was not correlated (data not shown) with the total root length regardless of the soil type, as was found in *B. juncea* and *B. napus* by Marschner et al. [11] who suggested that this correlation could be important for a plant´s capacity to exploit higher soil volume to reach more soil P. Our findings could be the result of harvesting older plants (ten-leaf) than in the mentioned study (six-leaf) when the correlation between P content and root length had the highest values, with a decreasing trend in older plants. This suggests that intensive root growth under low P availability is more relevant in young *Brassica* plants, while the other mechanisms to mobilize rhizosphere P are important in older plants. This is in contrast with findings on *B. napus,* which, when grown in deficient P, showed a positive at later growth stages, rather than during early development [21]. Moreover, we found weak correlation between plant P content and root DW (r = 0.41, *p* ≤ 0.05), and this parameter could be more important in later growth phases (older plants).

The *Brassica* genotypes used in this study differed in P use efficiency parameters regarding soil type (Table 3). The plants from RZ had the highest PUpE, PUtE and PUE values compared to other soils, showing that it was the most efficient soil in terms of P acquisition (uptake) and utilization, although plant P concentrations and content were very low. Kale genotypes are confirmed to have significantly different potential in P use efficiency, as has been found for the same genotypes in soilless cultivation [13]. This genotypic feature may be of interest for finding sustainable solutions in agriculture, since nutrient-efficient plants can produce higher yields per nutrient applied, or absorb more compared with other plants grown in similar agro-ecological conditions [26].

A difference in the PUE value between both genotypes was found, confirming the well-known genotypic variability of this parameter. Although the differences in PUpE and PUtE are obvious, questions remain as to which is more important for enhancing P efficiency [27]. The relative contribution of these parameters regarding soil type and genotype was analyzed by the methodology proposed by Moll et al. [28]. In soils with very low P availability, PUpE explained 96% (terra rossa) and 65% (rendzina) of variation in plant DW production (Table 4). Similarly, in another study, PUpE contribution was the highest in wheat grown on acid soils, and the same was found on calcareous soils, but in a smaller percentage [29]. Fluvisol, as a soil with P availability higher than the two other soils, saw PUE variation that was more greatly influenced by P utilization (PUtE = 60%), as was confirmed in the above mentioned study with wheat, as well as in fava beans [30] and maize [31]. Higher PupE for RZ, as the soil with the least available P, showed that uptake is more important than utilization under a low P supply for the used *Brassica* genotypes [13]. A high PUpE contribution in TR can be explained by significantly longer roots with a greater surface area. When comparing the root traits of maize hybrids in neutral and alkaline soil, it was found that the soil type affected the P acquisition efficiency, indicating the importance of soil properties [23].

The characterization of soil P pools by sequential–chemical fractionation methods is based on increasing the strength of P bound to soil inorganic and organic components: labile (NaOH/NH_4_Cl Pi), Fe-Al bound (CDB Pi), and Ca-bound (H_2_SO_4_ Pi) P in inorganic fractions, and labile (NaHCO_3_ Po), moderately labile (HCl Po), humic (NaOH/Po), and non-labile (H_2_SO_4_ Po) P for organic fractions. Labile Pi was not detected in TR (extraction with NH_4_Cl) or RZ (extraction with NaOH), while in FL this fraction (including Al-P) comprised 5% of total soil P (Figure 1A). Although this labile fraction was detected in a low concentration which could lead to the possible conclusion that it was not important for soil P movement, its values were found to remain relatively constant, as it was replenished from other fractions [32]. Similar to FL soil, low labile NaOH Pi concentrations were found in calcareous marsh soils from Spain [33] or subtropical soils of Iran [34]. The extraction of Fe- and Al-bound P in soils with high CaCO_3_ content was not expected, but an abundance of labile NaOH-Pi in RZ was expected [35]. Rose et al. [36] did not determine the depletion of labile Pi (as NaHCO3-Pi) in the fractionation of f soil types planted with wheat, canola and grain legume. However, the literature also revealed that this fraction is intensively used by plants [37,38]. For instance, *B. napus* uses this labile fraction, as was demonstrated at a lower concentration in the rhizosphere than in bulk soil [6]; therefore, we can assume that plants grown on FL uptake some labile Pi. Al-P was not found in TR, although additional extraction with NH_4_F was provided. In TR soil, most of the Pi was Fe-P (~50%), as it contains a high portion of Fe oxides [14]. This is also in agreement with findings that this fraction compromised most of the inorganic P in soil with lower pH/acid soil [39] and had a negative correlation between NaOH Pi, CaCO_3_, and pH [34].

Residual–soluble Pi extracted with complex CDB solution includes labile Ca-P, Fe, and Al-P reabsorbed from previous fractions and extracted with citrate-bicarbonate component, as well as P that is occluded in Fe oxides and released with strong reductant dithionite. CBD Pi fraction was determined in TR (~150 mg/kg) and FL (~100 mg/kg), and none in RZ (Figure 1A). Taking into account a high NaOH Pi (Fe-P) concentration and low CaCO_3_ content in TR, it is assumed that CDB Pi partially includes reabsorbed Fe-P from a previous fraction and P from Fe oxides. It is also assumed that labile Ca-P does not exist in this slightly acidic non-fertilized soil, as it has been found that only constant P fertilization can significantly affect this form [34]. In calcareous FL soil, CBD Pi can be mostly attributed to Ca-P, although the presence of Fe and Al-P is possible in occluded forms due to high soil pH. Ca-P from the CBD fraction is used intensively by plants, primarily Ca_2_-P, then Ca_8_-P, as was found in rice grown on calcareous soil and fertilized with NK [40]. Ca_2_-P depletion influenced the release of P from more fixed forms (Ca_8_-P). Otherwise, maize and oilseed rape grown in acid and neutral soil only depleted alkali-soluble Pi (NaHCO_3_ and NaOH-Pi) in rhizosphere soil, although the depletion of acid-soluble Pi (HCl-Pi) was not determined. A possible reason for this is that alkali-soluble Pi is adsorbed to exchangeable Fe/Al oxides/hydroxides surfaces, while HCl-Pi is almost non-exchangeable, except in highly alkaline soils [6].

Wang et al. [32] reported that NaOH Pi (complementary with our reductant soluble CBD Pi) decreased in the rhizosphere of legumes and wheat cultivated in alkaline and acid soils, while labile Pi did not change. Labile Pi was only detected in FL, indicating that a constant concentration is recovered after depletion with remobilization from CBD Pi. The last inorganic fraction extracted with strong acids, H_2_SO_4_ (TR) or HCl (RZ and FL), represents Ca-P in a form of lithogenic apatite (Ca_10_-P). It was found in all soils, in RZ only as a Pi fraction, and in the smallest amount in TR. The unusual findings of this fraction in TR may be attributed to the limestone parent rock. Increased P availability from Ca phosphates after solubilization by root-exudated organic acid anions (citric or malic) was reported for *Brassicas* [41]. In our RZ rhizosphere soil, we saw a pH decrease of 0.15–0.2 units (data not shown), which can result in a two- to five-fold increase in the solubility of Ca phosphates [42]. This acidification not only improves mineral P dissociation but also increases the mineralization of organic P sources [43].

It is considered that the organic fractions, labile NaHCO_3_-Po, and moderately labile HCl-Po are no less important for plant nutrition as two comparable and more available Pi fractions, and a plant’s ability to take these forms is connected with phosphatase activity in the rhizosphere [37]. In this study, organic phosphorus compromised 40–60% of total phosphorus, although with the highest portions of non-labile and highly resistant fractions, for humic NaOH-Po and non-labile H_2_SO_4_-Po (Figure 1B). Despite a lower presence of the two most labile organic P fractions in all three soils, organic P supported phosphatase activities regardless. The three studied soils significantly differed in AlPE and PDe activity, with the highest values in RZ which had the lowest Pi concentration. The activities of these two enzymes were the lowest in TR, despite it containing the highest portion of organic P in total P. Although without significant differences, AcPe was the highest in acidic TR as expected, but it was also present in all soils. However, two of the most labile Po fractions were not detected in TR, as they may have become less available by binding to metal oxides in the soil [32]. Organic P is primarily mineralized by soil microorganism’s activity in the rhizosphere, and so humic NaOH-Po can be used due to high AcPe activity. The activity of AcPE in FL and RZ was not significantly lower than in TR. In agreement with the present study, wheat grown in alkaline soils had AcPe activity four-fold higher in the rhizosphere than bulk soil, which influenced P uptake [44]. High AcPe and AlPe were also determined in the rhizosphere of alkaline soils, implying that the increase may be related to microbial activities [23]. Additionally, high enzymatic activity could be the result of release by microorganisms, the growth of which was stimulated with root exudates (e.g., organic acids anions) and was already noted as a possible reason for a decrease in pH.

Alkaline phosphatase was five- to six-fold higher in FL and RZ than in TR, which confirmed more intensive microorganism activity and their contribution to organic P mineralization, as this enzyme is only produced by microorganisms [45]. AlPe and DPe significantly positively correlated with HCl-Po, confirming their impact on more labile Po fractions. The DPe activities in our study were generally lower than AcPeacid and AlPe activities, as has also been determined as a general rule [46]. This is because the production of P monoesters from P diesters may stimulate the microbial synthesis of phosphomonoesterases [47].

NaOH-Po is P-bound on humic acids, and in FL it represents a stabile fraction, as this soil developed in Neretva Valley by fluvial sedimentation and seasonal flooding which stimulated the accumulation of organic matter [16]. After extensive reclamation in the last 50 years and an increased decomposition of organic matter in aerobic conditions, this P fraction was released to more labile organic and inorganic fractions, which can be used by plants (as was found in similar meliorated soils in Florida (USA)) [7]. The released P that remains in organo-metal compounds slows the precipitation of P as Ca phosphates or, in addition, the complexation of Ca with organic matter, and reduces the formation of Ca-phosphate minerals [48].

Organic P can be strongly adsorbed on ferric oxide surfaces in soils [3], which can be a reason for high humic and non-labile Po in TR, as was also confirmed by high correlations between these two organic and CBD Pi fractions.

## 4. Materials and Methods

### 4.1. Plant and Soil Materials

Two different *Brassica* species: Siberian kale—Red Russian (RR) (*B. napus* L. var. *napublaria*) and local kale—IJK 17 (*B. oleracea* L. var. *acephala*) were chosen for this study due to their different P use efficiency, as determined in a previous study [13].

The soils with low P availability used for the experiment in this study were collected from the 0–20 cm layer of abandoned agricultural land from three locations in the coastal part of Croatia: rendzina leptosol from Split (43°30′18″ N, 16°29′59″ E), terra rossa from Dugopolje (43°34′59″ N, 16°34′15″ E) and fluvisol eutric calcaric from Neretva Valley (42°59′40″ N, 17°31′54″ E). The main properties of the selected soils and available nutrient content are shown in Table 6. 

### 4.2. Experiment Set Up

The experiment was carried out in an unheated greenhouse at the Institute of Adriatic Crops in Split (43°31′ N. 16°27′ E. 50 m above sea level) during the autumn–winter season (2011/2012). A few seeds of kale genotypes were sown per cell of polystyrene plug trays in a mix of organic substrate and perlite 1:1 (*v*:*v*), and these were germinated in a growth chamber with a light/dark cycle of 14 h/10 h at 25 °C/20 °C and RH 80%/90% until the plants with cotyledons were about 5 cm in length. Then, they were thinned to one seedling per cell. Thereafter, the seedlings were grown in the greenhouse for 30 days followed by transplantation to 3 L plastic (polypropilene) pots filled with 3 kg of each soil after sieving. The pots were watered to maintain 70–80% of field capacity. Basal nutrients were added as nutrient solution to the soil at the following rates per pot: 300 mg of N, 200 mg of K (as KNO_3_ and NH_4_NO_3_), 7.5 mg of Mg (MgSO_4_·7H_2_O), 1.35 mg of B (H_3_BO_3_), 1.35 mg of Mn (MnCl_2_·4H_2_O), 0.15 mg of Zn (ZnSO_4_·7H_2_O), 0.6 mg of Cu (CuSO_4_·5H_2_O) and 0.006 mg of Mo ((NH_4_)_6_Mo_7_O_24_·4H_2_O). P fertilizer was not added to the soils. A few days after transplanting the solution with 2/3 N, all other elements were applied, and the rest of the N was applied two more times, 30 and 50 days after transplanting. In total, six treatments were set up (three soil types × two species), and the experiment was arranged as a randomized complete block design in four replications.

### 4.3. Plant Harvest and Measurements

Plants were harvested 70 days after sowing and divided into shoots and roots. Shoots were divided into leaves and stems. The leaf area was determined using a leaf area meter (LiCOR, Lincoln, NE, USA).

The roots were carefully removed with adhering soil that was stored for further analyses and was described as rhizosphere soil. After washing, the root samples were kept moist at 4 °C until root parameter measurements were performed. The samples were placed in a plexiglass tray filled with distilled water and placed on a scanner (Epson Pro, Nagano, Japan) to take images. Root images were analyzed with WinRHIZO Pro software (Regent Instruments, Sainte Foy, QC, Canada) to determine root length, surface area, volume and average root diameter. After the measurements, all the samples were oven dried at 70 °C for 48 h and weighed to measure the dry weight (DW).

For P analyses, the samples were ground and microwave-digested with HNO_3_ and H_2_O_2_. The total concentrations of the element were determined by inductively coupled plasma optical emission spectrometry (ICP-OES, Varian, Harbor City, CA, USA). Phosphorus content was calculated as a product of the P concentration and dry weight (DW) of plant parts. From root measurements and P content data, two additional variables were calculated:

1. Specific root length (SRL) (m root g^−1^ root DW);
2. Specific phosphorus uptake (SPU)—total plant P/total root length (mg P m^−1^).

All chemicals and reagents used were of adequate analytical grade and were purchased from Kemika (Zagreb, Croatia), Acros Organics (Geel, Belgium) and Sigma-Aldrich (St. Louis, MO, USA).

### 4.4. P Fractionation

In the rhizosphere soil samples, total P was determined, and P fractionation was performed by sequential extraction according to Kuo [51]. These procedures are based on the differential solubilities of the various inorganic P (Pi) forms. The phosphorus (P) fractionation scheme is showed in Figures S1 and S2. For non-calcareous soil (TR) ammonium chloride (NH_4_Cl) is first used to remove soluble and loosely bound P, followed by separating Al-P from Fe-P with NH_4_F. Fe-P was extracted with NaOH and the reductant-soluble P was removed via CDB (citrate-dithionite-bicarbonate) extraction. The Ca-P was removed with H_2_SO_4_ in non-calcareous soils or with HCl in calcareous soils (RZ and FL). In RZ and FL, soil soluble P was extracted with NaOH.

Organic phosphorus (Po) was fractionated by a modified scheme developed by Bowman and Cole [52]. Organic P in both calcareous and noncalcareous soils was fractionated into a labile pool (extracted with NaHCO_3_), a moderately labile pool (extracted with HCl), and a humic–acid pool (extracted with NaOH) (Figure S2). Finally, the highly resistant, nonlabile fraction was determined by ashing the residue from the NaOH extraction, followed by dissolution in H_2_SO_4_. The P concentration in all extracts was determined by ICP-OES.

### 4.5. Soil Enzymatic Analyses

The activity of AcPe, and AlPe, and DPe was determined on 1 g of wet weight of the rhizosphere soil, according to the method of Tabatabai [45]. Four replicates were sampled and measured for each of the six treatments (soil type × species), and each of them again in three measurement replicates.

For phosphomonoesterases, 0.2 mL of toluene, 4 mL of modified universal buffer (MUB) (pH 6.5 for assay of AcPe or pH 11 for assay of AlPe) and 1 mL of p-nitrophenyl phosphate solution (made in the same buffer) were added to the sample. After one hour of incubation at 37 °C, 1 mL of 0.5 M CaCl_2_ and 4 mL of 0.5 M NaOH were added, the flasks were shaken, and the soil suspension filtered through a Whatman no. 2 filter paper. The yellow color intensity was measured using a spectrophotometer at a wavelength of 420 nm, against the distilled water as a reference. A similar procedure was followed for phosphodiesterase activity, except that THAM buffer (Tris-hydroxymethyl-aminomethane, pH 8) was used instead of MUB before incubation, 0.1 M of THAM (pH 12) instead of NaOH after incubation, and bis-p-nitrophenyl phosphate instead of p-nitrophenyl phosphate.

The calibration curve for the p-nitrophenol content of the filtrate was plotted from the results obtained with standards containing 0, 10, 20, 30, 40, and 50 µg of (bis)p-nitrophenol, the same was done for AcPe, AlPe and DPe. Controls were performed with each soil sample to allow for color not derived from (bis)p-nitrophenol released by phosphatase activity.

### 4.6. P use Efficiency Parameters

P use efficiency (PUE) parameters were measured as P uptake efficiency (PUpE) and P utilization efficiency (PUtE). Both parameters were calculated as follows:

1. PupE = plant P content/soil available P (mg mg^−1^);
2. PutE = plant DW/P content (g DW mg P^−1^);

The relative contribution of variation in PUpE and PUtE in total PUE was analyzed as described by Moll et al. [28]. This methodology was initially presented for nitrogen but was adopted for available phosphorus from the soil supply [25]. It was developed to identify the contribution of two experimentally obtained variables (PUpE and PUtE) to the third one (PUE), which was obtained by multiplication. Logarithmic transformation was used to change PUE = PUpE × PUtE into log(PUE) = log(PUpE) + log (PUtE).

### 4.7. Statistical Analyses

To establish the main effects of the investigated factors and to determine possible interactions, the data were subjected to a two-way analysis of variance (ANOVA), with soils and species as factors. Means were compared using Tukey’s post hoc test at *p* < 0.05. For significant interactions, simple effects were computed at each level of the other factor using Tukey’s post hoc test at *p* < 0.05. The correlation coefficient values (r) were determined using treatment means. The statistical analysis was performed using StatView software (SAS institute, Cary, NC, USA).

## 5. Conclusions

The present study showed that leafy kale plants differed in root/shoot development when growing in three contrasting soils. Generally, a higher concentration of inorganic P influenced increased shoot growth, while in soils with a lower inorganic P pool both *Brassica* species saw more intensive root development. The data from this study suggest that different mechanisms of P uptake and use are activated in three contrasting soil types, and the species reacted similarly when grown in the same soil. The study showed how the same species enhance P uptake and utilization by changing root growth and physiological activities, which can be included as an important trait for future studies of genetic-environment interactions in low soil P conditions.

## Figures and Tables

**Table 1:** Shoot and root dry weight, leaf area, number of leaves, P concentration and P content of *Brassica* spp. grown in three soils for 70 days.

		Dry Weight (g Plant^−1^)			Root/Shoot Ratio		Leaf Area		Number of Leaves		P Concentration (mg g^−1^ DW)				P Content (mg Plant^−1^)			
		Shoot		Root			cm^2^ Plant^−1^		Plant^−1^		Shoot		Root		Shoot		Root	
	Terra rossa	2.00 ± 0.20	b	0.26 ± 0.02	0.14 ± 0.02	a	267 ± 36	b	8.2 ± 0.39	b	1.02 ± 0.08	b	1.25 ± 0.07	b	2.12 ± 0.35	b	0.33 ± 0.03	b
Soil	Rendzina	1.60 ± 0.08	b	0.20 ± 0.02	0.13 ± 0.01	a	214 ± 20	b	7.7 ± 0.18	b	0.87 ± 0.04	b	1.29 ± 0.03	b	1.39 ± 0.10	b	0.26 ± 0.03	b
	Fluvisol	3.45 ± 0.31	a	0.26 ± 0.04	0.07 ± 0.01	b	543 ± 59	a	10.5 ± 0.35	a	1.85 ± 0.11	a	2.77 ± 0.14	a	6.35 ± 0.74	a	0.71 ± 0.12	a
Genotype	RR	2.10 ± 0.32		0.26 ± 0.02	0.14 ± 0.01	a	272 ± 41	b	9.0 ± 0.48		1.30 ± 0.13		1.67 ± 0.19		3.05 ± 0.70		0.44 ± 0.06	
	IJK17	2.55 ± 0.26		0.22 ± 0.03	0.09 ± 0.01	b	410 ± 54	a	8.6 ± 0.39		1.24 ± 0.16		1.86 ± 0.25		3.52 ± 0.75		0.43 ± 0.10	

The results are expressed as means (±SE) with different letters in columns differing significantly (*p* < 0.05) according to Tukey’s test.

**Table 2:** Root length, diameter, specific root length (SRL), and specific P uptake (SPU) of *Brassica* spp. grown in three soils for 70 days, as well as soil enzyme activity at harvest.

										Enzyme Activity						
		Root Length		Root Diameter		SRL		SPU		Acid Phosphatase		Alkaline Phosphatase		Phospho-Diesterase		
		m Plant^−1^		mm		m g^−1^		mg P m^−1^		mg PNP kg^−1^ h^−1^						
	Terra rossa	25.43 ± 2.2	a	0.38 ± 0.02	a	98.3 ± 7.5	a	0.11 ± 0.02	b	153 ± 9		51.7 ± 11.1	b	19.2 ± 3.3	c	
Soil	Rendzina	17.01 ± 1.1	b	0.32 ± 0.01	b	89.8 ± 8.6	ab	0.10 ± 0.01	b	124 ± 26		280 ± 28.5	a	127 ± 9.9	a	
	Fluvisol	18.00 ± 1.9	b	0.34 ± 0.01	ab	68.3 ± 4.1	b	0.39 ± 0.05	a	82.7 ± 10		264 ± 13.1	a	87.7 ± 9.9	b	
Genotype	RR	21.13 ± 1.7		0.35 ±0.02		81.0 ± 8.5		0.21 ± 0.06		116 ± 20		181 ± 31.3		78.9 ± 14		
	IJK17	19.16 ± 1.8		0.34 ±0.01		91.9 ± 4.7		0.20 ± 0.04		116 ± 15		234 ± 37.3		78.1 ± 15		

The results are expressed as means (±SE) with different letters in columns differing significantly (*p* < 0.05) according to Tukey´s test.

**Table 3:** P uptake efficiency (PUpE), P utilization efficiency (PUtE) and P use efficiency (PUE) of two *Brassica* genotypes grown in three soils.

		PUpE		PUtE		PUE	
		mg P mg^−1^P		g DW mg P^−1^			
	Terra rossa	0.55 ± 0.09	b	0.99 ± 0.08	a	0.51 ± 0.05	b
Soil	Rendzina	2.03 ± 0.14	a	1.13 ± 0.05	a	2.25 ± 0.10	a
	Fluvisol	0.43 ± 0.04	b	0.55 ± 0.04	b	0.23 ± 0.02	c
Genotype	RR	0.83 ± 0.20		0.93 ± 0.10		0.77 ± 0.23	b
	IJK17	1.01 ± 0.22		0.90 ± 0.08		0.91 ± 0.28	a

The results are expressed as means (±SE) with different letters in columns differing significantly (*p* < 0.05) according to Tukey’s test.

**Table 4:** Contribution of PUpE and PUtE variability to shoot DW.

PUE Component	Variability in Shoot DW Production		
	Soil		
	Terra rossa	Rendzina	Fluvisol
PUpE	0.96	0.65	0.40
PUtE	0.04	0.35	0.60
	Genotype		
	IJK 17	RR	
PUpE	0.72	0.64	
PUtE	0.28	0.36	

PUpE—P uptake efficiency, PUtE—P utilization efficiency.

**Table 5:** Correlation coefficients between enzymatic activity and fractions and total organic P in rhizosphere soil of *Brassica* genotypes.

	Acid Phosphatase	Alkaline Phosphatase	Phospho-Diesterase	NaHCO_3_-Po	HCl-Po	NaOH-Po	H_2_SO_4_-Po	Total Po
Acid phosphatase		−0.06	−0.16	−0.37	−0.52 *	0.41	0.47 *	0.43
Alkaline phosphatase			0.75 ***	0.3	0.69 **	−0.88 ***	−0.88 ***	−0.89 ***
Phospho- diesterase				0.36	0.54 *	−0.84 ***	−0.80 ***	−0.84 ***

*** Significant at *p* < 0.001; ** significant at *p* < 0.01; * significant at *p* < 0.05.

**Table 6:** Selected soil properties.

Soil	pH_H2O_	pH_KCl_	CaCO_3_	CaO	TOC	Total N	Available P *	Total P ^#^	Available K ^¥^	Texture
			g kg^−1^					mg kg^−1^		
Terra rossa	6.70	5.63	8.0	-	4.8	0.12	5.0	833	79	Clay
Rendzina	8.35	7.31	616	193	17.5	0.32	0.9	373	129	Silt loam
Fluvisol	8.16	7.70	356	486	32.7	0.69	18	408	198	Sandy loam

* Available P was determined after extraction with (NH_4_)_2_SO_4_ [17] via the Troug method. ^#^ Total P was determined by soil digestion in *aqua regia* [49]. ^¥^ Available K was determined after extraction with NH_4_-acetate [50]. Total N—total nitrogen determined by Kjeldahl method.

## Data Availability

Not applicable.

## Supplementary Materials

The following supporting information can be downloaded at: https://www.mdpi.com/article/10.3390/plants12061295/s1, Figure S1: Scheme of inorganic P fractionation; Figure S2: Scheme of organic P fractionation.

## References

[1] Cordell D., Drangert J.O., White S. (2009). The story of phosphorus: Global food security and food for thought. Glob. Environ. Chang..

[2] Nadeem M., Wu J., Ghaffari H., Kedir A.J., Saleem S., Mollier A., Singh J., Cheema M. (2022). Understanding the adaptive mechanisms of plants to enhance phosphorus use efficiency on podzolic soils in boreal agroecosystem. Front. Plant Sci..

[3] Celi L., Barberis E., Turner B.L., Frossard E., Baldwin D.S. (2005). Abiotic Stabilization of Organic Phosphorus in the Environment. Organic Phosphorus in the Environment.

[4] Machado C.T.D.T., Furlani Â.M.C. (2004). Root phosphatase activity, plant growth and phosphorus accumulation of maize genotypes. Sci. Agric..

[5] Hedley M.J., Stewart J.W.B., Chauhan B.C. (1982). Changes in inorganic and organic soil phosphorus fractions induce by cultivation practices and by laboratory incubation. Soil Sci. Soc. Am. J..

[6] Cabeza R.A., Myint K., Steingrobe B., Stritsis C., Schulze J., Claassen N. (2017). Phosphorus fractions depletion in the rhizosphere of young and adult maize and oilseed rape plants. J. Soil Sci. Plant Nutr..

[7] Castillo M.S., Wright A.L. (2008). Soil phosphorus pools for Histosols under sugarcane and pasture in the Everglades, USA. Geoderma.

[8] Richardson A.E., Hocking P.J., Simpson R.J., George T.S. (2009). Plant mechanisms to optimise access to soil phosphorus. Crop Pasture Sci..

[9] Manzoor A., Dippold M.A., Loeppmann S., Blagodatskaya E. (2022). Two-phase conceptual framework of phosphatase activity and phosphorus bioavailability. Front. Plant Sci..

[10] Fernández M.C., Belinque H., Boem F.G., Rubio G. (2009). Compared phosphorus efficiency in soybean, sunflower and maize. J. Plant Nutr..

[11] Marschner P., Solaiman Z., Rengel Z. (2007). Brassica genotypes differ in growth, phosphorus uptake and rhizosphere properties under P-limiting conditions. Soil Biol. Biochem..

[12] Schwerdtner U., Lacher U., Spohn M. (2022). Soy and mustard effectively mobilize phosphorus from inorganic and organic sources. Nutr. Cycl. Agroecosyst..

[13] Urlić B., Dumičić G., Goreta Ban S., Romić M. (2016). Phosphorus-use efficiency of kale genotypes from coastal Croatia. J. Plant Nutr..

[14] Durn G. (2003). Terra Rossa in the Mediterranean Region: Parent Materials, Composition and Origin. Geol. Croat..

[15] FAO (2014). World Reference Base for Soil Resources.

[16] Romić D., Romić M., Zovko M., Bakić H., Ondrašek G. (2012). Trace metals in the coastal soils developed from estuarine floodplain sediments in the Croatian Mediterranean region. Environ. Geochem. Health.

[17] Bolland M.D.A., Kumar V., Gilkes R.J. (1994). A comparison of five soil phosphorus tests for five crop species for soil previously fertilized with superphosphate and rock phosphate. Fertil. Res..

[18] Hammond J.P., Broadley M.R., White P.J., King G.J., Bowen H.C., Hayden R., Meacham M.C., Mead A., Overs T., Spracklen W.P. (2009). Shoot yield drives phosphorus use efficiency in Brassica oleracea and correlates with root architecture traits. J. Exp. Bot..

[19] Niu Y.F., Chai R.S., Jin G.L., Wang H., Tang C.X., Zhang Y.S. (2013). Responses of root architecture development to low phosphorus availability: A review. Ann. Bot..

[20] Vengavasi K., Pandey R., Soumya P.R., Hawkesford M.J., Siddique K.H. (2021). Below-ground physiological processes enhancing phosphorus acquisition in plants. Plant Physiol. Rep..

[21] Duan X., Jin K., Ding G., Wang C., Cai H., Wang S., White P.J., Xu F., Shi L. (2020). The impact of different morphological and biochemical root traits on phosphorus acquisition and seed yield of Brassica napus. Field Crops Res..

[22] Robles-Aguilar A.A., Pang J., Postma J.A., Schrey S.D., Lambers H., Jablonowski N.D. (2019). The effect of pH on morphological and physiological root traits of *Lupinus angustifolius* treated with struviteg as a recycled phosphorus source. Plant Soil.

[23] Erel R., Bérard A., Capowiez L., Doussan C., Arnal D., Souche G., Gavaland A., Fritz C., Eric J., Visser W. (2017). Soil type determines how root and rhizosphere traits relate to phosphorus acquisition in field-grown maize genotypes. Plant Soil.

[24] Morrow de la Riva L. (2010). Root Etiolation as a Strategy for Phosphorus Acquisition in Common Bean. Master’s Thesis.

[25] Lyu Y., Tang H., Li H., Zhang F., Rengel Z., Whalley W.R., Shen J. (2016). Major crop species show differential balance between root morphological and physiological responses to variable phosphorus supply. Front. Plant Sci..

[26] Fageria N.K., Baligar V.C., Li Y.C. (2008). The role of nutrient efficient plants in improving crop yields in the twenty first century. J. Plant Nutr..

[27] Wang X., Shen J., Liao H. (2010). Acquisition or utilization, which is more critical for enhancing phosphorus efficiency in modern crops?. Plant Sci..

[28] Moll R.H., Kamprath E.J., Jackson W.A. (1982). Analysis and interpretation of factors which contribute to efficiency of nitrogen utilization. Agron. J..

[29] Manske G.G.B., Ortiz-Monasterio J.I., van Ginkel M., González R.M., Fischer R.A., Rajaram S., Vlek P.L.G. (2001). Importance of P uptake efficiency versus P utilization for wheat yield in acid and calcareous soils in Mexico. Eur. J. Agron..

[30] Daoui K., Karrou M., Mrabet R., Fatemi Z., Draye X., Ledent J.F. (2012). Genotypic variation of phosphorus use efficiency among Moroccan faba bean varieties (*Vicia faba major*) under rainfed conditions. J. Plant Nutr..

[31] Parentoni S.N., Souza Júnior C.L.D. (2008). Phosphorus acquisition and internal utilization efficiency in tropical maize genotypes. Pesqui. Agropecu. Bras..

[32] Wang Y., Marschner P., Zhang F. (2012). Phosphorus pools and other soil properties in the rhizosphere of wheat and legumes growing in three soils in monoculture or as a mixture of wheat and legume. Plant Soil..

[33] Jiménez-Cárceles F.J., Álvarez-Rogel J. (2008). Phosphorus fractionation and distribution in salt marsh soils affected by mine wastes and eutrophicated water: A case study in SE Spain. Geoderma.

[34] Adhami E., Owliaie H.R., Molavi R., Rezaei Rashti M., Esfandbod M. (2013). Effects of soil properties on phosphorus fractions in subtropical soils of Iran. J. Soil Sci. Plant Nutr..

[35] Williams J.D.H., Syers J.K., Harris R.F., Armstrong D.E. (1971). Fractionation of inorganic phosphate in calcareous lake sediments. Soil Sci. Soc. Am. Proc..

[36] Rose T.J., Hardiputra B., Rengel Z. (2010). Wheat, canola and grain legume access to soil phosphorus fractions differs in soils with contrasting phosphorus dynamics. Plant Soil.

[37] Nuruzzaman M., Lambers H., Bolland M.D., Veneklaas E.J. (2006). Distribution of carboxylates and acid phosphatase and depletion of different phosphorus fractions in the rhizosphere of a cereal and three grain legumes. Plant Soil.

[38] Vu D.T., Tang C., Armstrong R.D. (2008). Changes and availability of P fractions following 65 years of P application to a calcareous soil in a Mediterranean climate. Plant Soil.

[39] Ruiz J.M., Delgado A., Torrent J. (1997). Iron-related phosphorus in over fertilized European soils. J. Environ. Qual..

[40] Shen J., Li R., Zhang F., Fan J., Tang C., Rengel Z. (2004). Crop yields, soil fertility and phosphorus fractions in response to long-term fertilization under the rice monoculture system on a calcareous soil. Field Crops Res..

[41] Hoffland E. (1992). Quantitative evaluation of the role of organic acid exudation in the mobilization of rock phosphate by rape. Plant Soil.

[42] Solaiman Z., Marschner P., Wang D., Rengel Z. (2007). Growth, P uptake and rhizosphere properties of wheat and canola genotypes in an alkaline soil with low P availability. Biol. Fertil. Soils.

[43] Neumann G., Romheld V. (1999). Root excretion of carboxylic acids and protons in phosphorus-deficient plants. Plant Soil.

[44] Marschner P., Solaiman Z., Rengel Z. (2005). Growth, phosphorus uptake, and rhizosphere microbial community composition of a phosphorus defficient wheat cultivar in soils differing in pH. J. Plant Nutr. Soil Sci..

[45] Tabatabai M.A. (1994). Soil enzymes. Methods of Soil Analysis, Part 2—Microbiological and Biochemical Properties.

[46] Criquet S., Braud A., Nèble S. (2007). Short-term effects of sewage sludge application on phosphatase activities and available P fractions in Mediterranean soils. Soil Biol. Biochem..

[47] Turner B.L., Haygarth P.M. (2005). Phosphatase activity in temperate pasture soils: Potential regulation of labile organic phosphorus turnover by phosphodiesterase activity. Sci. Total Environ..

[48] Halajnia A., Haghnia G.H., Fotovat A., Khorasani R. (2009). Phosphorus fractions in calcareous soils amended with P fertilizer and cattle manure. Geoderma.

[49] (1995). Soil Quality—Extraction of Trace Elements Soluble in Aqua Regia.

[50] Jones J.B. (2001). Laboratory Guide for Conducting Soil Tests and Plant Analysis.

[51] Kuo S., Sparks D.L. (1994). Phosphorus. Methods of Soil Analysis.

[52] Bowman R.A., Cole C.V. (1978). An exploratory method for fractionation of organic phosphorus from grassland soils. Soil Sci..

