# Supplementary material for: Phosphorus Use Efficiency of Leafy Brassica sp. Grown in Three Contrasting Soils: Growth, Enzyme Activity and Phosphorus Fractionation
Source: Plants (Basel). 2023 Mar 13;12(6):1295. doi: 10.3390/plants12061295 (PMC10056877; doi:10.3390/plants12061295)
Supplement: Supplementary file 1 [file plants-12-01295-s001.zip › plants-2185579-supplementary.pdf]

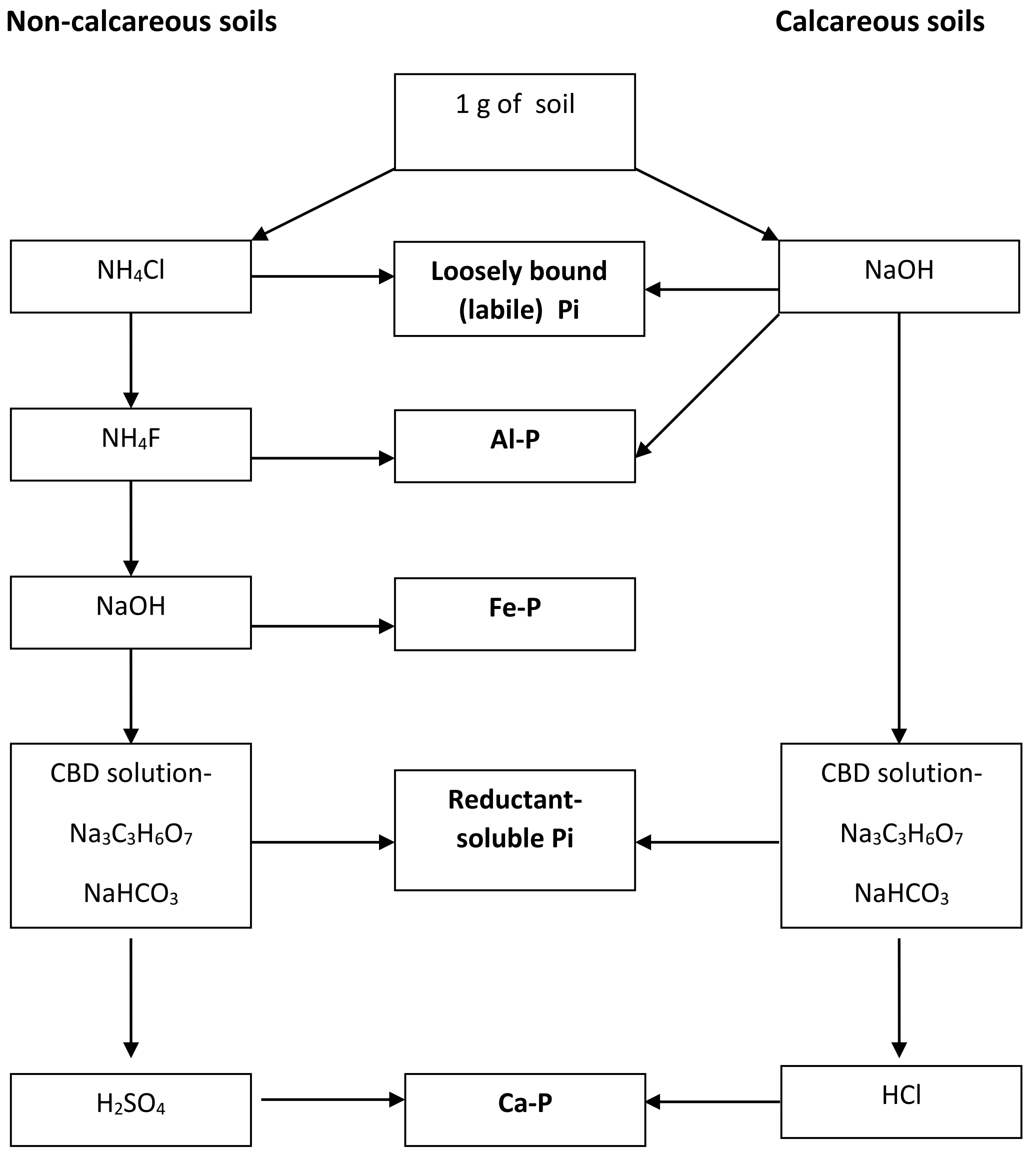

Figure S1. Scheme of inorganic P fractionation

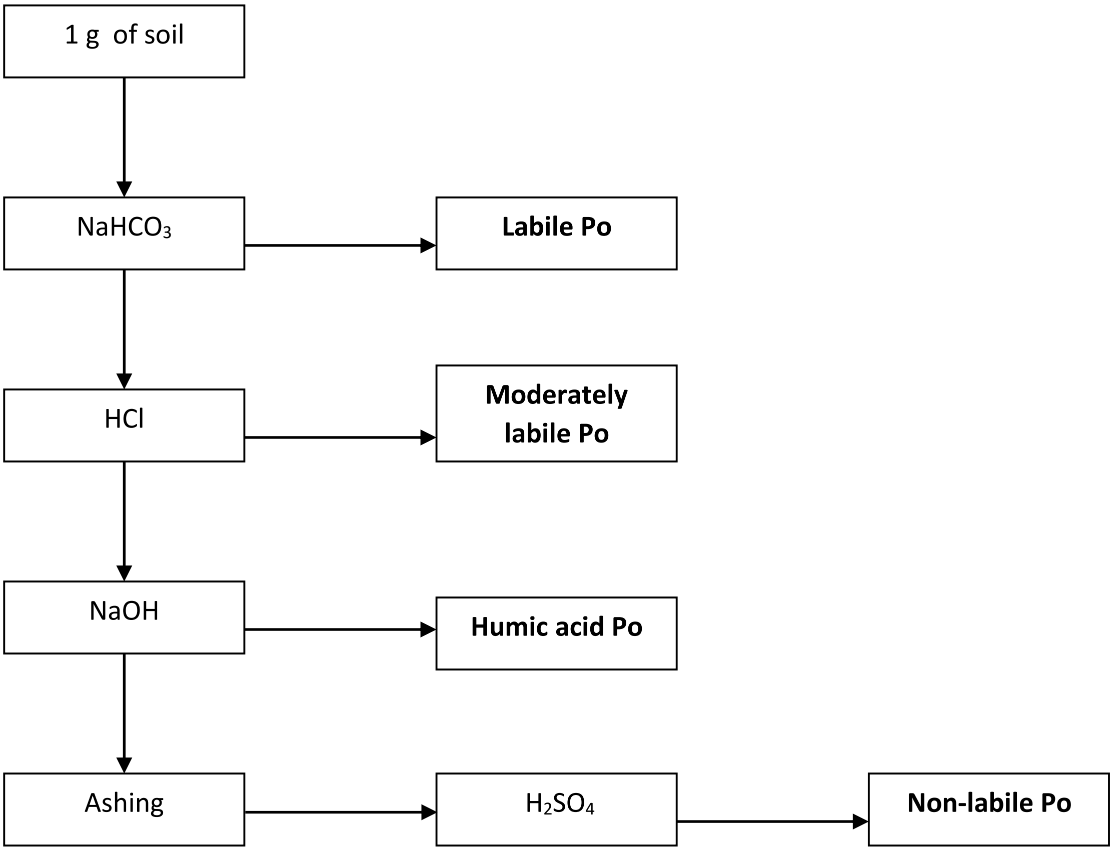

Figure S2. Scheme of organic P fractionation
